# Mercury: Forest Fire Fallout

**DOI:** 10.1289/ehp.115-a21a

**Published:** 2007-01

**Authors:** David A. Taylor

In the summer of 2005, wildfires raged over 3.4 million hectares of Alaska and Canada’s northern boreal forests, according to combined figures from the Canadian Large Fire Database and the Alaska Large Fires Database. It was the region’s second worst fire season on record. The worst was the year before, when 5.7 million hectares burned. The number of very large “megafires” in the circumpolar region is increasing, says Merritt Turetsky, an assistant professor in the Department of Plant Biology at Michigan State University. But alongside the obvious hazards posed by smoke and flames is one perhaps unexpected risk: emissions of mercury (Hg) released from the peat that is relatively common in these northernmost forests.

About 80% of the world’s peat-land is located at high latitudes. Peat soils absorb more Hg than other soils because the Hg is buried by the accumulating peat after it falls to the soil surface. This Hg can then be transformed to methyl Hg (MeHg), which accumulates as it goes up the food chain. The EPA has deemed MeHg a possible carcinogen and set a limit of 2 ppb Hg for drinking water; the FDA put the limit for MeHg in seafood at 1 ppb.

Because they are far from human populations, boreal forest fires can burn for weeks before they are reported, and are allowed to burn longer than their southerly counterparts. “We’ve seen more than a doubling of burn area per year since the 1950s,” says Turetsky. Dry seasons being up to a month longer than in the past—partly a result of climate change—are a big factor, Turetsky and coauthors argue in volume 33, number 16 (2006) of *Geophysical Research Letters*. The authors estimate that as the scale of the fires increases, exacerbated by projected global warming, the impact of Hg on the food chain also will increase.

“Any changes in carbon cycling will influence the release of this mercury to the environment,” notes Richard Bindler, an assistant professor of ecology and environmental science at Umeå University, Sweden. “The real health concern lies in the aquatic food chain and the concentrations of mercury in the fish we eat.”

Turetsky began to notice high rates of MeHg in her work on carbon storage. She and coauthor Jennifer Harden have quantified organic matter and Hg concentrations in samples of frozen peat cored from sites across western Canada. Their samples, taken from sites that varied in forest canopy, soil drainage, and forest age, were augmented with comparable samples recorded in a database of organic matter storage across the boreal region. They used a 20-year record of the extent and timing of fires and a simple fire emission model to estimate how much of this stored Hg could potentially be released into the atmosphere as a result of wildfire activity across western Canada. They found Hg emissions 15 times higher than previous estimates, which had not accounted for peat’s ability to store Hg.

“Many peatlands in interior North America are forested and have very dense soils,” says Turetsky. “This forested peat can store a lot of carbon from the atmosphere, which is good, and it can store a lot of mercury from the atmosphere as well, which is also good—until these fire emissions occur.” The emissions affect both the atmosphere and runoff into northern lakes and streams.

Turetsky and colleagues have gone a step further than previous emissions models, says Bindler, who calls their model and ideas “conceptually very sound.” For him, a question remains, though: to what extent do the study’s estimates of soil Hg represent other boreal forests?

Turetsky hopes the study will call attention to the startling increase of wildfires across northern North America. “We really should be paying attention to growing toxicities in the north as well,” she says. “In the Great Lakes, where I am, we catch lots of salmon. Increasingly, those catches carry mercury warnings.”

## Figures and Tables

**Figure:**
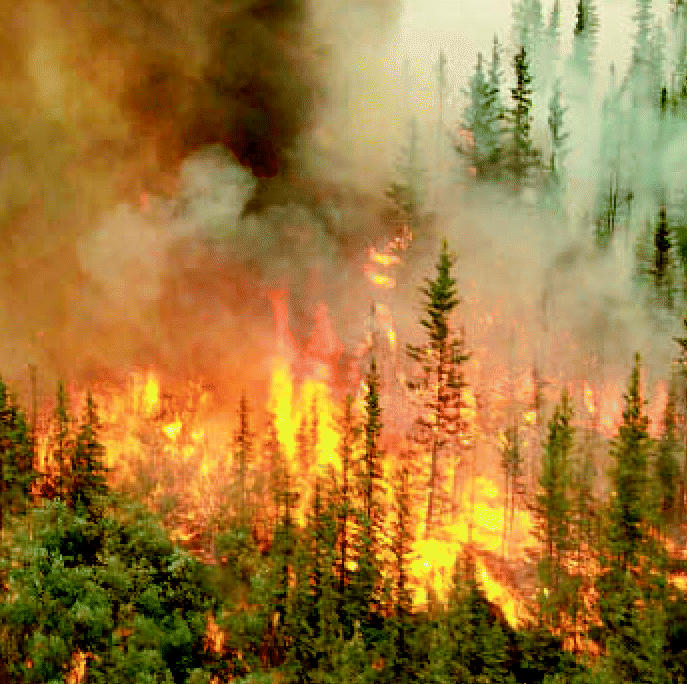
For peat’s sake. Peatlands in boreal forests act as natural sponges of atmospheric mercury. Significant amounts of mercury are thus released when such forests burn, a more frequent occurrence due to climate change.

